# Printers’ Diseases

**Published:** 1860-12

**Authors:** 


### PRINTERS' DISEASES.

Many printers are in the habit of holding types between their teeth. When the types are damp, and especially when they are new, a substance is upon their surface which, when applied to the lips, causes troublesome fissures, which sometimes end in incurable cancers, which eat life away by piecemeals in the slow process of weary months.

This same substance sometimes finds its way to the inner side of the lips by means of the tongue and the saliva, causing troublesome tumors, which inflame, ulcerate, and rapidly assume the form of torturing cancer. The only remedy is prevention, by keeping the type out of the mouth. The most common of all diseases among printers are those of the air-passages, of which bronchitis is the most frequent. Next to that, inflammation of the lungs and consumption, in consequence of the bent position of their bodies, which prevents full, deep breathing, when the lungs from inaction become debilitated, and unable to resist impressions from cold, to which printers are so liable, in consequence of their rooms being kept very warm, and their inattention to proper rules when they leave them. Being so much in the composing-room they become forgetful of the cold without, and at the close of the day, in that tired, weary condition that follows a ten hours' labor, they come out on the street, stand around the office-doors talking with one another and looking around, and before they are aware of it, they are often chilled through, and thus, through mere inattention, the foundation is laid for the fatal ailments enumerated. Nearly one fourth of printers die of consumptive forms of disease. Hernia is common, especially among pressmen. Dimness of sight, short-sightedness and weakness of eyes, are very common, in consequence of the constant strain on that organ, and its exposure to artificial light. Fissures and hard lumps often form on the forefinger and thumb of the right hand from handling damp type. But the great disease which sweeps so many of them into a premature grave is consumption, but which would not occur with a tithe of the frequency if the following few precautions were habitually taken:

First, regularity in eating and in bodily habits. Second, put on all the extra clothing before going into the street, avoid stopping an instant, but move on at a brisk pace with the mouth closed, so that instead of a dash of cold air going in upon the lungs at each breath to chill them, it may be first warmed, by being compelled to pass around through the nostrils.

